# Evaluation of the Antitumor Effects of Platinum-Based [Pt(*η*^1^-C_2_H_4_-OR)(DMSO)(phen)]^+^ (R = Me, Et) Cationic Organometallic Complexes on Chemoresistant Pancreatic Cancer Cell Lines

**DOI:** 10.1155/2023/5564624

**Published:** 2023-09-11

**Authors:** Erika Stefàno, Luca Giulio Cossa, Federica De Castro, Erik De Luca, Viviana Vergaro, Giulia My, Gianluca Rovito, Danilo Migoni, Antonella Muscella, Santo Marsigliante, Michele Benedetti, Francesco Paolo Fanizzi

**Affiliations:** Department of Biological and Environmental Sciences and Technologies (DiSTeBA), University of Salento, Via Monteroni, I-73100 Lecce, Italy

## Abstract

Pancreatic cancer is one of the most lethal malignancies with an increasing incidence and a high mortality rate, due to its rapid progression, invasiveness, and resistance to anticancer therapies. In this work, we evaluated the antiproliferative and antimigratory activities of the two organometallic compounds, [Pt(*η*^1^-C_2_H_4_-OMe)(DMSO)(phen)]Cl (**1**) and [Pt(*η*^1^-C_2_H_4_-OEt)(DMSO)(phen)]Cl (**2**), on three human pancreatic ductal adenocarcinoma cell lines with different sensitivity to cisplatin (Mia PaCa-2, PANC-1, and YAPC). The two cationic analogues showed superimposable antiproliferative effects on the tested cells, without significant differences depending on alkyl chain length (Me or Et). On the other hand, they demonstrated to be more effective than cisplatin, especially on YAPC cancer cells. For the interesting cytotoxic activity observed on YAPC, further biological assays were performed, on this cancer cell line, to evaluate the apoptotic and antimetastatic properties of the considered platinum compounds (**1** and **2**). The cytotoxicity of **1** and **2** compounds appeared to be related to their intracellular accumulation, which was much faster than that of cisplatin. Both **1** and **2** compounds significantly induced apoptosis and cell cycle arrest, with a high accumulation of sub-G1 phase cells, compared to cisplatin. Moreover, phenanthroline-containing complexes caused a rapid loss of mitochondria membrane potential, ΔΨ_*M*_, if compared to cisplatin, probably due to their cationic and lipophilic properties. On 3D tumor spheroids, **1** and **2** significantly reduced migrated area more than cisplatin, confirming an antimetastatic ability.

## 1. Introduction

Despite its relatively low incidence, pancreatic cancer is one of the most lethal malignancies with a high mortality rate due to its invasiveness, rapid progression, and resistance to treatments [1]. Pancreatic cancer is currently the seventh leading cause of cancer-related death in the world after lung, colon, liver, stomach, breast, and esophagus cancers [2], both in males and females, but it is expected to become the third in the next years [3, 4]. The prognosis in patients with pancreatic tumors is the poorest of any common solid malignancy, with a 5-year overall survival of about 10% [5]. Most patients are diagnosed with advanced stage, and half of them are characterized by metastases [6]. The nonspecific symptoms associated with pancreatic cancer make the diagnosis difficult, and the important blood vessels in proximity to the tumor can be easily invaded [7, 8]. Since the pancreas is a multifunctional organ, it consists of different cell types, from which several kinds of pancreatic tumors are derived. More generally, pancreatic tumors can be either exocrine (derived by duct cells and acinar cells) or endocrine (e.g., *β* cells) [9]. Pancreatic ductal adenocarcinoma (PDAC) and its variants represent 90% of all pancreatic carcinomas; the remaining part includes nonductal tumors, such as acinar cells and neuroendocrine tumors [7]. The pathophysiology of pancreatic adenocarcinoma is characterized by a multistep genetic alteration, which involves oncogenes that are responsible for its initiation and progression, including KRAS, CDKN2A, TP53, and SMAD4 [10].

Nowadays, gemcitabine is the first-line therapy for pancreatic cancer, approved by the U.S. Food and Drug Administration (FDA) in 1996 [11]. On the other side, cisplatin is one of the most effective and widely used chemotherapy drugs, being able to induce apoptosis in pancreatic cancer cells [12–14]. It was observed that a combination of gemcitabine and cisplatin can enhance DNA damage and improve survival in patients with pancreatic cancer [6, 15]. However, severe side effects and resistance phenomena often occur after cisplatin treatment, still leaving this interesting research field open and aiming to obtain new platinum-based compounds with an improved pharmacokinetic and pharmacodynamic profile and reduced adverse effects [16–18].

Recently, we obtained the [Pt(*η*^1^-C_2_H_4_-OMe)Cl(phen)] complex [19], through the formation of a pentacoordinate intermediate [20], starting from phenanthroline and Zeise's salt in basic methanol. From this precursor, we synthesized and characterized the new platinum complex, [Pt(*η*^1^-C_2_H_4_-OMe)(DMSO)(phen)]^+^ (**1**), demonstrating high antiproliferative effects on various human cancer cell lines [21]. Particularly, on neuroblastoma cells, we observed a fast induction of mitochondrial apoptotic process and antimigratory capacity with respect to cisplatin [22]. Furthermore, ^1^H-NMR-based metabolomics analyses allowed us to highlight an early alteration of glutathione (L-*γ*-glutamyl-L-cysteinyl-glycine; GSH) metabolism pathway compared to cisplatin [23], making this complex able to bypass cancer drug resistance related to GSH antioxidant system [24]. We also observed that the cytotoxic activity of this phenanthroline-containing compound can be optimized by modifying alkyl chain length since antiproliferative properties of platinum complexes can be influenced by molecular lipophilicity. In particular, we synthesized the new cationic [Pt(*η*^1^-C_2_H_4_-OR)(DMSO)(phen)]^+^ analogues {R = Me (**1**), Et (**2**), Pr (**3**), Bu (**4**)} and observed that complex **2** with the Et moiety resulted to be generally more cytotoxic in the tested series of complexes. This allowed us to hypothesize that an optimal activity can be obtained for specific chain lengths [25].

**Figure 1:**
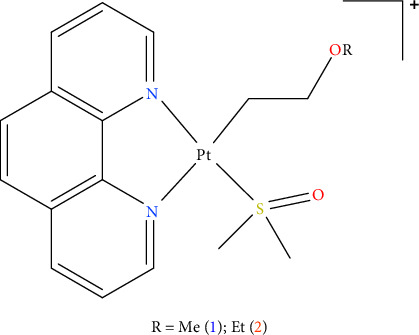
Chemical structure of [Pt(*η*^1^-C_2_H_4_-OR)(DMSO)(phen)]^+^, R = methyl (**1**), ethyl (**2**).

This work aims to compare the effect of cisplatin and two of the phenanthroline-containing complexes, [Pt(*η*^1^-C_2_H_4_-OMe)(DMSO)(phen)]^+^ (**1**) and [Pt(*η*^1^-C_2_H_4_-OEt)(DMSO)(phen)]^+^ (**2**), Figure 1, on three human pancreatic ductal adenocarcinoma cell lines which were chosen for their differing sensitivity to cisplatin (Mia PaCa-2, PANC-1, and YAPC) [26–30] in order to investigate their antiproliferative properties and their ability to induce specific cell death processes.

## 2. Materials and Methods

### 2.1. Synthesis of Complexes

Commercially available reagents and solvents were used as received, without further purification. Cisplatin was supplied by the Sigma-Aldrich Chemical Company. The [PtCl(*η*^1^-C_2_H_4_OR)(phen)] (R = Me, Et) complexes were synthesized according to a previously reported procedure and gave satisfactory analytical data [25].

All NMR measurements were performed on a Bruker Avance DPX 400 NMR spectrometer or a Bruker AVANCE III 600 Ascend NMR spectrometer (Bruker, Ettlingen, Germany), equipped with a TCI cryoprobe incorporating a *z*-axis gradient coil and automatic tuning/matching, at 300 K. ^1^H NMR monodimensional spectra and [^1^H, ^195^Pt]-HETCOR bidimensional experiments were recorded by using deuterated CDCl_3_ or D_2_O as solvents. ^1^H NMR spectra were referenced to TMS; the residual proton signal of the solvent [CDCl_3_; *δ*(^1^H) = 7.24 ppm; D_2_O; *δ*(^1^H) = 4.7 ppm] was used as the internal standard. ^195^Pt NMR chemical shifts were referenced to H_2_[PtCl_6_] [*δ* (^195^Pt) = 0 ppm] in D_2_O, as the external reference.

### 2.2. Cell Cultures

YAPC (DSMZ, Braunschweig, Germany) cells were cultured in RPMI 1640 medium (EuroClone, Pero, MI) supplemented with 10% (vol/vol) heat-inactivated fetal bovine serum (FBS), glutamine 2 mM, penicillin (100 U/mL), and streptomycin (100 mg/mL). MIA PaCa-2 and PANC-1 cells (ATCC, Rockville, MD) were cultured in Dulbecco's Modified Eagle Medium (DMEM) (4,5 mg/L glucose) (EuroClone, Pero, MI) supplemented with 10% (vol/vol) heat-inactivated FBS, glutamine 2 mM, penicillin (100 U/mL), and streptomycin (100 mg/mL). Cells were grown in a humidified incubator containing 5% CO_2_ in air at 37°C and used for biological assays when 70–80% confluence was reached.

### 2.3. Cell Viability Assay

Pancreatic cancer cell viability was measured with sulforhodamine B (SRB) colorimetric assay. 100 *μ*L of a cell suspension was added to each well of a 96-well microtiter plate (10^4^ cells per well). After overnight incubation, cells were treated with different concentrations of cisplatin, **1** and **2** for 24 and 48 h. At that time, 100 *μ*L of ice-cold 10% (wt/vol) trichloroacetic acid was added to each well for 30 min at 4°C. After that, the plates were washed five times with double distilled water and air-dried overnight. 70 *μ*L of 0.4% (wt/vol) SRB solution was added to each well and incubated for 30 min, followed by four washes with 1% (vol/vol) acetic acid. Finally, SRB was dissolved in 200 *μ*L of 10 mM unbuffered Tris base solution, and color intensity was measured fluorometrically at 560 nm. The percentage of cell survival was calculated as the absorbance ratio of treated to vehicle-treated control cells. The data presented are means ± standard deviation from eight replicates of three independent experiments.

### 2.4. Analysis by ICP-AES (Inductively Coupled Plasma Atomic Emission Spectroscopy)

For the determination of platinum concentration, cells were incubated with 30 and 50 *μ*M, respectively, of Pt(II) compounds for 1, 6, and 18 h. After the incubation time, the cellular pellet of each sample was recovered and treated with 1 mL of 67% super-pure nitric acid at room temperature for 24 h. Then, samples were diluted to a final volume of 5 mL, to obtain a suitable concentration of the acid, filtered, and analyzed, as previously reported [21]. The platinum concentration in the analyzed samples was determined by a Thermo iCAP 6000 spectrometer. The spectrophotometer was calibrated with a calibration line consisting of four points, each corresponding to a concentration of the element: 1, 10, 100, and 1000 g/L.

### 2.5. Cell Cycle Analysis

The effect of the Pt(II) complexes on the cell cycle was evaluated using the nuclear staining dye propidium iodide (PI) (Thermo Fisher Scientific Inc.) that is frequently used. After treatment, cells were washed twice in PBS and harvested by trypsinization. Then, cells were fixed in 70% cold ethanol and kept overnight at 20°C prior to staining. After fixation, cells were centrifuged at 2000 rpm for 5 min, and ethanol was removed. Finally, cells were resuspended in 0.5 mL 0.05% Triton X-100/PBS staining solution containing 20 *μ*g/mL PI and 2 *μ*g/mL RNase. Data acquisition and analyses were performed by flow cytometer (BD Biosciences, San Jose, CA, USA), and cycle distribution (sub-G1, G0/G1, S, and G2/M phase fraction) was analyzed using BD Accuri C6 Software.

### 2.6. Apoptosis Analysis

#### 2.6.1. Annexin V-FITC/PI Assay

Cell apoptosis was determined by using the Annexin V-fluorescein isothiocyanate (FITC) kit (Thermo Fisher Scientific Inc.). After exposure to Pt(II) compounds at the IC_50_ concentration for 24 h, YAPC cells were collected, washed twice with PBS, and subjected to centrifugation at 1200 × g for 5 min at room temperature. Subsequently, the cell pellet was resuspended in Annexin V-FITC and propidium iodide (PI) solutions. After incubation for 15 min at room temperature in the dark, additional Annexin V binding buffer (10 mM Hepes, 140 mM NaCl, and 2.5 mM CaCl_2_) was added to each sample. The cells were analyzed using a flow cytometer (BD Biosciences, San Jose, CA, USA) and BD Accuri C6 Software.

#### 2.6.2. Measurement of Mitochondrial Membrane Potential

Pancreatic cells were seeded and, after reaching 70–80% confluence, incubated with JC-1 (Enzo Life Sciences, Farmingdale, NY, USA) for 15 min (37°C, 5% CO_2_). Then, cells (∼2 × 10^6^) were washed twice in PBS and resuspended in a cuvette containing 2 mL Krebs-Ringer Hepes (KRH) buffer (140 mmol/L NaCl, 5 mmol/L KCl, 2 mmol/L CaCl_2_, 1 mmol/L MgCl_2_, 10 mmol/L Hepes, 6 mmol/L glucose, pH 7.4). Cells were exposed to 50 *μ*M cisplatin, **1** or **2**, and transferred to Jasco FP-750 Spectrofluorometer (JASCO Corporation, Tokyo, Japan). JC-1 fluorescence was assessed at different times (0, 5, 15, 30, and 45 min) in each experiment. For each experiment and each time point, the ratio of JC-1 fluorescence intensity at 590 nm and at 520 nm was calculated and used as a qualitative estimate of ΔΨ_M_. Data are expressed as the treatment-induced change in the 590/520 nm fluorescence ratio relative to the initial (control) 590/520 nm ratio.

#### 2.6.3. Evaluation of Cell Apoptosis Using Fluorescence Microscopy

The apoptotic effect of Pt(II) complexes on YAPC cells was established by fluorescence microscopy, by combining JC-1 and DAPI (4,6-diamin-2-phenylindole) staining. PDAC cells were incubated with Pt(II) complexes for 24 h and, after treatment, stained with JC-1 according to the protocol illustrated in the previous paragraph. Subsequently, cells were fixed in 4% (wt/vol) paraformaldehyde and stained with 1 *μ*g/mL DAPI (Thermo Scientific, Rockford, USA) in PBS for 40 min. Cells were mounted on glass slides, covered, and analyzed using an EVOS XL Cell Imaging System microscope (Thermo Fisher, Waltham, MA, USA). The reversible changes in JC-1 aggregation were measured by Image 1.62 software (National Institutes of Health, Bethesda, MD, USA).

### 2.7. 3D Spheroid-Based Migration Assay

A suspension of 2.5 × 10^5^ cells/mL was used to form tumor spheroids as previously reported [31]. After 4 days of incubation, tumor spheroids were transferred in 96-well plates (one tumor spheroid per well, in a final volume of 100 *μ*L). After waiting for the tumor spheroids to adhere to the plate bottom (about 1 h), cells were treated with sublethal concentrations of cisplatin, **1** and **2** complexes. Images were obtained at 24 h, using an inverted microscope. The area covered by the cells that migrated from the spheroids was measured using PhotoShop C6 software (Adobe). Data were normalized to the original size of each spheroid recorded at *t*0 [formula: (migrated area at *t* = *x*/migrated area at *t* = 0) × 100].

### 2.8. Statistical Analyses

Statistical analyses were performed with GraphPad Prism 8 software (GraphPad Software, San Diego, CA, USA). Experimental points represent means ± S.D. for each experimental group. The normality of data before the analyses was confirmed by the Kolmogorov–Smirnov tests. Statistical analysis was carried out using ANOVA associated with Tukey's multiple comparisons test. A *p* value <0.05 was considered to achieve statistical significance.

## 3. Results

### 3.1. [Pt(*η*^1^-C_2_H_4_-OMe)(DMSO)(phen)]^+^ (**1**) and [Pt(*η*^1^-C_2_H_4_-OEt)(DMSO)(phen)]^+^ (**2**) Inhibition of Cell Growth

The cytotoxicity of the two analogue [Pt(*η*^1^-C_2_H_4_-OR)(DMSO)(phen)]^+^ (R = Me, **1**; Et, **2**) complexes was evaluated by SRB assay on three human PDAC (Mia PaCa-2, PANC-1, and YAPC) to better understand, besides differences with respect to cisplatin, the possible role of alkyl chain length on inhibition of cell proliferation. IC_50_ values (concentration required for 50% growth inhibition) were calculated after 24 and 48 h of incubation with Pt(II) complexes.

**Figure 2:**
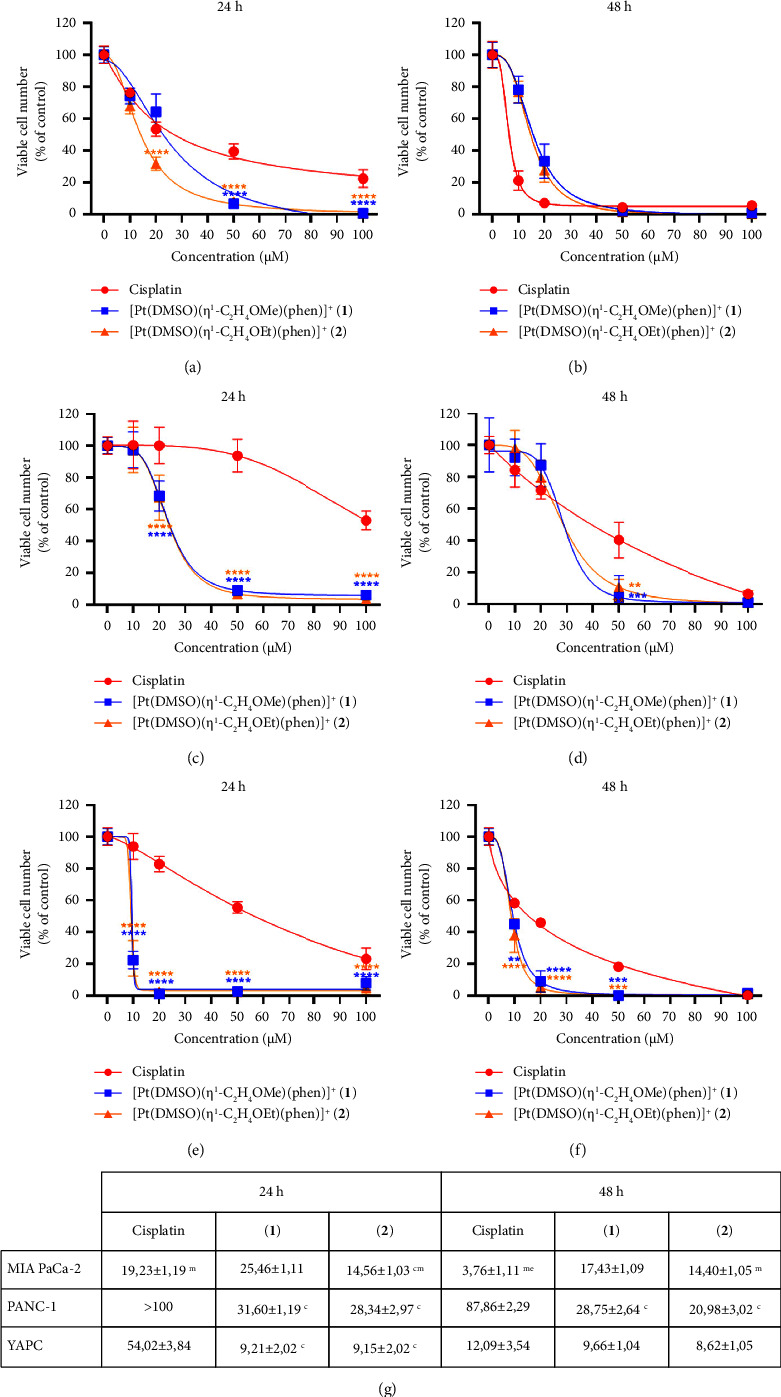
Cytotoxic effects of cisplatin, [Pt(*η*^1^-C_2_H_4_-OMe)(DMSO)(phen)]^+^ (**1**), and [Pt(*η*^1^-C_2_H_4_-OEt)(DMSO)(phen)]^+^ (**2**). Complexes **1** and **2** were tested at 0–100 *μ*M concentrations on (a, b) MIA PaCa-2, (c, d) PANC-1, and (e, f) YAPC cells and compared with cisplatin after 24 and 48 h incubations. Viable cell numbers were determined by SRB assay. Asterisks (^*∗*^*p* < 0.05; ^*∗∗*^*p* < 0.01; ^*∗∗∗*^*p* < 0.001; ^*∗∗∗∗*^*p* < 0.0001) indicate values that are significantly lower than those of cisplatin at the same concentration and time point. Data represent the mean ± standard deviation for four different experiments run in eight replicates, expressed as a percentage of control. (g) IC_50_ values were calculated and presented as means ± standard deviation. Letters indicate IC_50_ values that are significantly lower (*p* < 0.05) than those of cisplatin (^c^), **1** (^m^), and **2** (^e^) at the same time point.

Exposure of PDAC cells to Pt compounds at concentrations ranging from 0 to 100 *μ*M resulted in a concentration-dependent increase in cell death, as shown in Figure 2. Both cisplatin and phen-containing complexes significantly inhibited pancreatic cell viability, but in a different way, depending on cell line type, as shown in Figures 2(a)–2(g). In general, **1** and **2** induced a higher cytotoxic effect, especially at shorter incubation times, than cisplatin in the examined cell lines, with an IC_50_ between 9.15 ± 2.02 and 31.6 ± 1.19 *μ*M after 24 h of treatment, as shown in Figure 2(g). Cisplatin resulted in higher cytotoxicity only for MIA PaCa-2 cells (IC_50_ = 3.76 ± 1.11 *μ*M) after 48 h of incubation, compared to **1** and **2** complexes, as shown in Figures 2(b) and 2(g). Interestingly, the two [Pt(*η*^1^-C_2_H_4_-OR)(DMSO)(phen)]^+^ complexes demonstrated to be highly effective in PANC-1 cells, which appeared to be the most cisplatin-resistant cells among the tested cell lines (IC_50_ > 100 after 24 h; IC_50_ = 87.86 ± 2.29 *μ*M after 48 h).

[Pt(*η*^1^-C_2_H_4_-OMe)(DMSO)(phen)]^+^ (**1**) and [Pt(*η*^1^-C_2_H_4_-OEt)(DMSO)(phen)]^+^ (**2**) showed a similar cytotoxic effect in all cell lines after 48 h of incubation (*p* > 0.05). Complex **2** was significantly more cytotoxic than complex **1** only in MIA PaCa-2 cells, but only up to 24 h of treatment, as shown in Figures 2(a), 2(g). Thus, generally, the two phen-containing complexes did not differ in their antiproliferative activity on the tested PDAC lines. Instead, **1** and **2** showed a higher cytotoxicity, also at shorter incubation times in the three pancreatic cell lines, compared to cisplatin.

Considering the cytotoxicity assay results, we decided to further investigate the cell death process induction, caused by **1** and **2** complexes with respect to cisplatin, on YAPC cells, due to the observed markedly different responses, as shown in Figures 2(e)–2(g).

### 3.2. Intracellular Accumulation of Pt(II) Complexes

**Figure 3:**
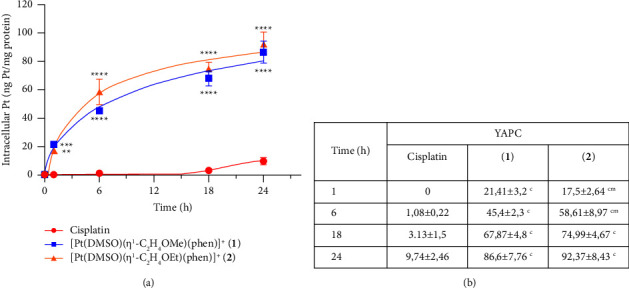
Intracellular uptake after [Pt(*η*^1^-C_2_H_4_-OMe)(DMSO)(phen)]^+^ (**1**), [Pt(*η*^1^-C_2_H_4_-OEt)(DMSO)(phen)]^+^ (**2**), and cisplatin treatments in YAPC cell lines. (a, b) YAPC cells were continuously exposed to 50 *μ*M of cisplatin, **1**, and **2**, for 1, 6, 18, and 24 h. Total intracellular accumulation was determined by inductively coupled plasma atomic emission spectroscopy (ICP-AES). Each point represents the mean ± S.D. of three different experiments and is indicated as ng of Pt(II)/mg of protein. Asterisks indicate values that are significantly different (^*∗*^*p* < 0.05; ^*∗∗*^*p* < 0.01; ^*∗∗∗*^*p* < 0.001; ^*∗∗∗∗*^*p* < 0.0001) from those of cisplatin at the same time point. (b) Letters indicate values that are significantly higher (*p* < 0.05) than those of cisplatin (^c^), **1** (^m^), and **2** (^e^) at the same concentration and time point.

Total intracellular Pt(II) content was evaluated in YAPC, by ICP-AES, after incubation with 50 *μ*M (IC_50_ value calculated after treatment with cisplatin) of cisplatin, **1**, and **2** for 1, 6, 18, and 24 h. As already noted for [Pt(*η*^1^-C_2_H_4_-OMe)(DMSO)(phen)]^+^ (**1**) [21], [Pt(*η*^1^-C_2_H_4_-OEt)(DMSO)(phen)]^+^ (**2**) seemed to enter cells via a passive or facilitated passive transport very quickly. The intracellular accumulation of **1** and **2** complexes was much higher than cisplatin starting from 1 h of incubation (*p* < 0.05), as shown in Figure 3. During the first 1–6 h of treatment, complex **2** accumulation in treated cells was also slightly higher, if compared to complex **1**, but both complexes showed comparable accumulation of platinum values after 18 h, as shown in Figure 3(b). Therefore, these two Pt(II) complexes exhibited very similar uptake profiles and cytotoxicity levels in YAPC cells, suggesting that the cytotoxicity of complexes **1** and **2** on YAPC cells after 24 h of incubation may be related to a high intracellular uptake with respect to cisplatin (about 9-fold), as shown in Figures 2 and 3.

### 3.3. [Pt(*η*^1^-C_2_H_4_-OEt)(DMSO)(phen)]^+^ (**2**) Induces Apoptosis and Causes Cell Cycle Arrest in YAPC Cells

**Figure 4:**
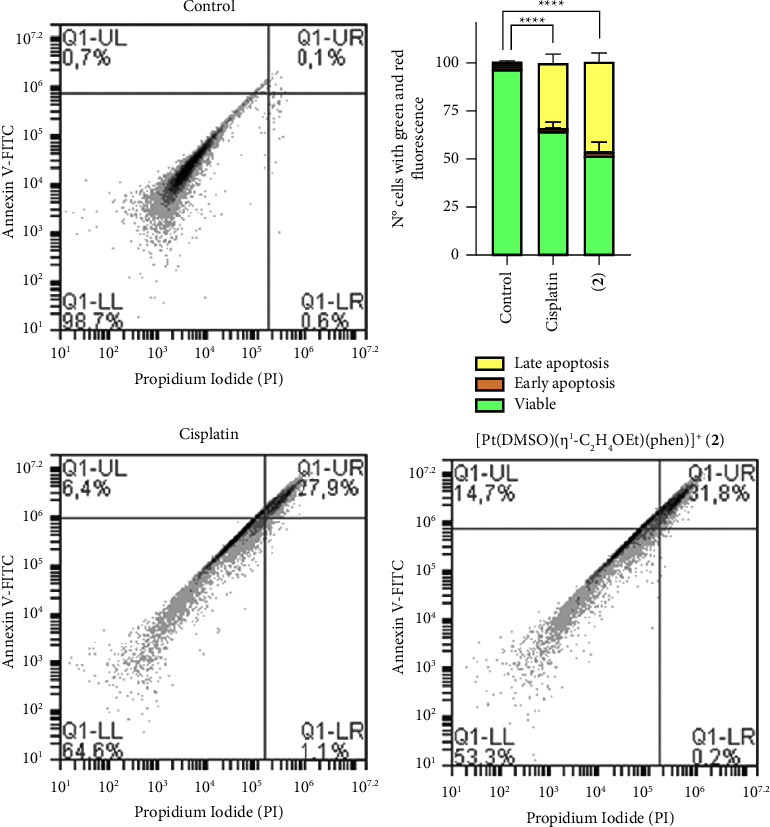
[Pt(*η*^1^-C_2_H_4_-OEt)(DMSO)(phen)]^+^ (**2**) and cisplatin-induced apoptosis in YAPC cells. Cell death was quantified by flow cytometry (BD Accuri C6 flow cytometer) after annexin V-fluorescein isothiocyanate (FITC)/propidium iodide (PI) staining. YAPC cells were treated or not with 50 *µ*M cisplatin and complex **2** for 18 h. Q1-UL, PI+ (cells undergoing necrosis); Q1-UR, annexin V-FITC + PI+ (cells in the late period of apoptosis and undergoing secondary necrosis); Q1-LR, annexin V-FITC + PI− (cells in the early period of apoptosis); Q1-LL, annexin V-FITC − PI− (living cells). The percentage of viable and dead cells was quantified using BD Accuri C6 software and displayed as a bar graph. The images are representative of three independent experiments. Asterisks (^*∗∗∗∗*^*p* < 0.0001) indicate values of viable cells that are significantly different from the control after treatment with cisplatin and complex **2**.

**Figure 5:**
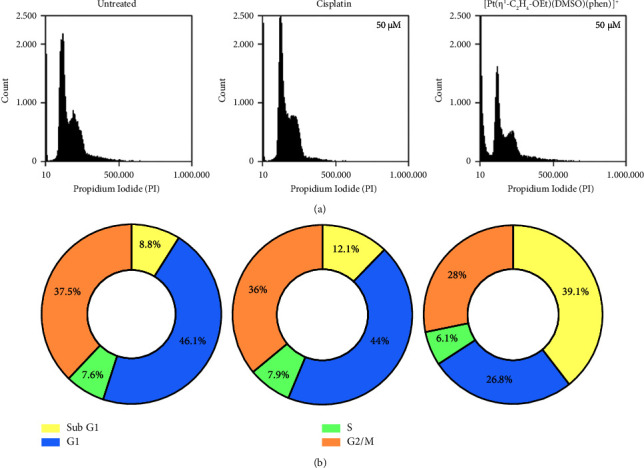
Effects of cisplatin and [Pt(*η*^1^-C_2_H_4_-OEt)(DMSO)(phen)]^+^ (**2**) on the cell cycle. (a) Flow cytometry (BD Accuri C6 flow cytometer) was used to investigate the cell cycle distribution of propidium iodide-stained cells after treatment with YAPC with or without cisplatin and complex **2** for 18 hours. (b) The pie charts indicate the percentages of cells in the G1, S, or G2/M phases of the cell cycle. These images are representative of three independent experiments.

Cell death after exposure to cisplatin, **1**, and **2** complexes was also investigated by flow cytometry, measuring the percentage of cells that exhibited Annexin V-FITC and/or PI fluorescence. Since complexes **1** and **2** gave comparable results, only flow cytometry results obtained after treatment with **2** are shown in Figures 4 and 5.

YAPC cells were exposed to phen-containing complexes to assess the apoptosis induction compared to the control and cisplatin-treated, as shown in Figure 4. Incubation with cisplatin and [Pt(*η*^1^-C_2_H_4_-OEt)(DMSO)(phen)]^+^ (2) resulted in significant apoptosis enhancement compared to the untreated cells (*p* < 0.0001). In addition, complex **2** induced cell death more than cisplatin after 18 h of treatment (*p* < 0.05). In the control group, only 1.4% apoptosis was observed while 35.4 and 46.7% apoptosis and necrosis were found in the groups treated with 50 *μ*M cisplatin and **2**, respectively. The influence of Pt complexes on YAPC cell proliferation was evaluated by flow cytometry in PI (propidium iodide)-stained cells after treatment for 18 h. Here, the classical antineoplastic drug cisplatin was used as positive controls.

G1 represents the longest phase of the cell cycle; thus, the largest fraction of cells is usually in the G1 phase. The sub-G1 method for death cell detection is based on the principle that after DNA endonucleolytic cleavage, low-molecular-weight DNA fragments are released from cells during prolonged fixation. That will yield a population of cells that bind a quantitative DNA stain (PI) to a lesser extent than what is characteristic of G1 cells [32]. In YAPC cells, [Pt(*η*^1^-C_2_H_4_-OEt)(DMSO)(phen)]^+^ (**2**) caused a significant and time-dependent (data not shown) increase in the percentage of YAPC cells in the sub-G1 and decrease in the percentage of cells in G2/M, compared to cisplatin, as shown in Figures 4(a) and 4(b).

### 3.4. Determination of Mitochondrial Changes Using Fluorescent JC-1 Staining and Fluorimetry

Mitochondria play an important role in metabolism, free radical generation, and cell death. We wanted to determine whether [Pt(*η*^1^-C_2_H_4_-OEt)(DMSO)(phen)]^+^ (**2**) and cisplatin induced the loss or disruption of mitochondrial transmembrane potential (ΔΨ_*M*_) in YAPC cells using the cation dye JC-1. Mitochondrial membrane potential is an important parameter of mitochondrial function, acting as an indicator of cell health [33]. In healthy cells (high ΔΨ_*M*_), JC-1 forms J-aggregate complexes with intense red fluorescence. Instead, in cells with low ΔΨ_*M*_, JC-1 remains in the monomeric form, which exhibits green fluorescence. Depending on whether JC-1 exists as a monomer or J-aggregate, it emits at 520 nm (green) and 590 nm (red), respectively, after excitation at 488 nm [33, 34]. The red/green fluorescence intensity ratio indicates the change in ΔΨ_*M*_ and then the occurrence of apoptosis.

**Figure 6:**
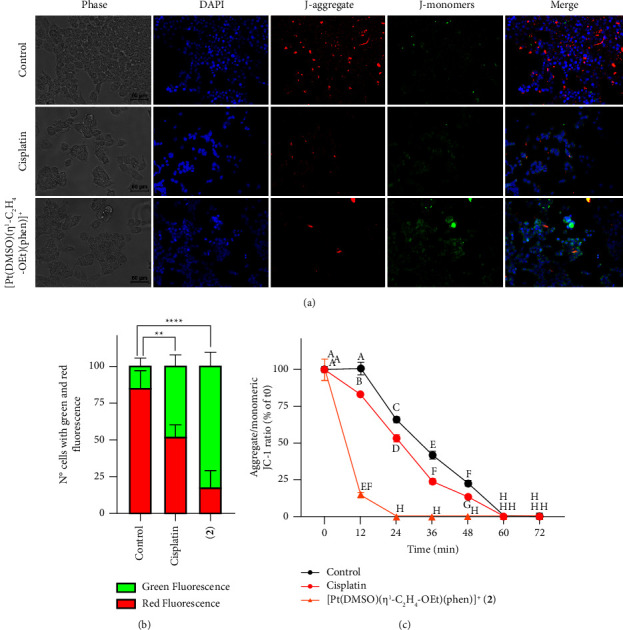
Analysis of mitochondrial membrane potential using the cationic dye JC-1 in YAPC cells. (a) Double staining of nuclei (DAPI) and mitochondria (JC-1) was evaluated in YAPC cells by fluorescence microscopy (scale bar: 60 *μ*m). (a, b) Cells were incubated or not with 50 *μ*M cisplatin and complex 2 for 24 hours and stained with DAPI and JC-1 dyes. (b) Quantification of cells observed with green and red fluorescence (expressed as a percentage) was carried out using Image J. Data are represented as the mean ± SD from three independent experiments. Asterisks (^*∗∗*^*p* < 0.01; ^*∗∗∗∗*^*p* < 0.0001) indicate values of green fluorescence that are significantly higher than untreated cells. (c) The sums of J-aggregate and J-monomer fluorescence from the measurements were obtained from spectrophotometry data and were used for calculating the respective JC-1 ratios. The results represent the mean ± SD from three independent measurements. Values with shared letters are not significantly different according to Tukey's multiple comparisons test.

To observe the shift in fluorescence emission of JC-1, cancer cells were treated or not with 50 *μ*M of Pt complexes for 24 h and stained with the fluorescence probe.  Figure 6(a) shows images obtained using fluorescence microscopy. The quantification of cells with green and red fluorescence is displayed as a bar graph, as shown in Figure 6(c). Red fluorescence, the sign of preserved ΔΨ_*M*_, was observed in almost all untreated controls (about 85%), whereas YAPC cells incubated with Pt compounds displayed green fluorescent signals, index of mitochondrial membrane depolarization, as shown in Figures 6(a) and 6(c). The exposure to [Pt(*η*^1^-C_2_H_4_-OEt)(DMSO)(phen)]^+^ (**2**) increased of about 6-fold the cells with green fluorescence emission compared to control (*p* < 0.0001), and about 3-fold with respect to cisplatin (*p* < 0.01), as shown in Figure 6(b). Superimposable effects were observed when cells were exposed to complex **1** (data not shown).

Mitochondrial membrane depolarization (ΔΨ_*M*_) was also detected fluorometrically by a shift in fluorescence emission of the cationic probe JC-1. YAPC cells were stained with JC-1 and then incubated with cisplatin, **1** and **2** complexes, and the shift in fluorescence emission of JC-1 was followed for 12 to 72 min. Also, in this case, results obtained after treatment with the two phenanthroline-containing complexes were similar (*p* > 0.05). The aggregate/monomeric JC-1 ratio is comparable in **1** and **2** complexes (only results regarding **2** are shown in Figure 6). Fluorometric analysis showed that treatment of YAPC cells with [Pt(*η*^1^-C_2_H_4_-OEt)(DMSO)(phen)]^+^ (**2**) caused an early transition of JC-1 fluorescence, with a shift toward green fluorescence that was detected as early as the first minutes after exposure to Pt complex, as shown in Figure 6(c). ΔΨ_*M*_ significantly decreased 12 min after the addition of complex **2** and reached a minimum after 24 min, as shown in Figure 6(c). Differently from phenanthroline-containing complexes, cisplatin treatment decreased ΔΨ_*M*_ similar to **2** only 1 h after incubation, as shown in Figure 6(c).

### 3.5. [Pt(*η*^1^-C_2_H_4_-OEt)(DMSO)(phen)]^+^ (**2**) Reduced YAPC Cells Migration/Invasion

**Figure 7:**
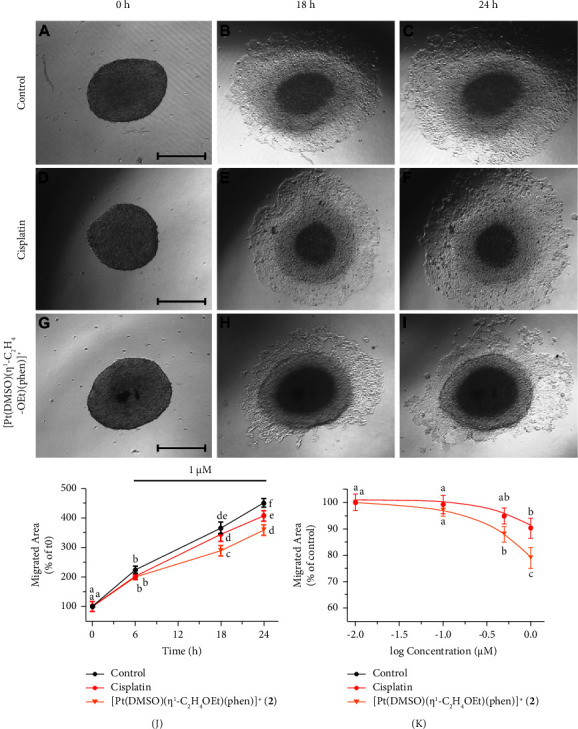
Effects of cisplatin and [Pt(*η*^1^-C_2_H_4_-OEt)(DMSO)(phen)]^+^ (**2**) on cell migration. (A-K) Tumor spheroids were transferred into a 96-well flat-bottomed migration plate and treated with 0.1, 0.5, 1.0, and 1.5 *μ*M [Pt(*η*^1^-C_2_H_4_-OEt)(DMSO)(phen)]^+^ (**2**) or cisplatin for 24 h. Digital images of the spheroids were then captured at 0, 6, 18, and 24 h (scale bar = 300 *μ*m). (A–J) Tumor spheroids treated or not treated with 1 *μ*M cisplatin and 2 at 6, 18, and 24 h. (J) Migrating areas were measured and reported on the graph as a percentage of *t*0. (K) Tumor spheroids treated or not treated with 0.1 to 1 *μ*M cisplatin and 2 at 24 h. Migrating areas were measured and reported on the graph as a percentage of the control. (J,K) All data were expressed as the mean ± standard deviation (SD) values of three independent experiments. Values with shared letters are not significantly different according to post-hoc Tukey's test.

The inhibition by [Pt(*η*^1^-C_2_H_4_-OEt)(DMSO)(phen)]^+^ (**2**) of pancreatic YAPC cells' migration processes was evaluated by the 3D tumor spheroid-based assay. Sublethal concentrations of cisplatin and **2** complexes, which did not significantly induce cytotoxicity in YAPC cells, were used for the treatment (0.1 to 1 *μ*M). The effects of 1 *µ*M of Pt complexes after different incubation times are shown in Figures 7(A)–7(J). [Pt(*η*^1^-C_2_H_4_-OEt)(DMSO)(phen)]^+^ (**2**) significantly reduced cell migration in a time- (Figure 7(J)) and concentration-dependent (Figure 7(K)) manner, starting from a concentration of 0.5 *µ*M (*p* < 0.01). After a treatment of 24 h, the tumor-spheroids' migration area was reduced by 10% (*p* < 0.01) and 20% (*p* < 0.001) after treatment with cisplatin and complex **2**, respectively, as shown in Figure 7(J).

## 4. Discussion and Conclusions

Pancreatic cancer is one of the most lethal malignancies, with a rising incidence and a high mortality rate. According to GLOBOCAN estimates of cancer incidence and mortality worldwide (produced by the International Agency for Research on Cancer) in 2020, pancreatic cancer accounts for 496,000 cases with 466,000 deaths [2]. The high mortality associated with pancreatic cancer is due to its rapid progression, invasiveness, and resistance to current treatments [1]. Pancreatic ductal adenocarcinoma (PDAC) is the most common pancreatic cancer type, with a 5-year survival rate of less than 5% [5]. The high mortality rate is often related to late diagnosis, and the aggressive nature of malignant cells disseminate to nearby tissues at an early stage of the disease, making treatment difficult [35]. Cisplatin is one of the best chemotherapeutic drugs and the first metal-based drug, which has demonstrated a high efficacy in the treatment of various cancers (testicular, ovarian, head and neck, bladder, lung, cervical cancer, melanoma, lymphomas, and others) [36]. The use of cisplatin for PDAC treatment in combination with chemotherapy was evaluated in several clinical trials, but the response was limited by intrinsic and acquired drug resistance [37]. This latter can be developed by cancer cells in different ways: (i) reduced accumulation due to increased extrusion of cisplatin by transporters; (ii) sequestration and inactivation by GSH and other cytoplasmic scavengers with nucleophilic properties; (iii) reduced DNA damage recognition and apoptotic response; (iv) other unspecific adaptive responses resulting in strong antiapoptosis ability and resistance of cancer cells to cisplatin (e.g., dysregulation of TP54, MAPK, PI3K/AKT, NF-*κ*B, and Stat3 pathways) [38].

Organometallic complexes containing 1,10-phenanthroline (phen) showed high antitumoral activity against cisplatin-resistant cells, thanks to the capability of phenanthroline to interact with DNA and intercalate between nucleic acid base pairs, inhibiting DNA synthesis and DNA repair systems [39–42]. Moreover, intercalating agents can induce cytotoxicity by inhibiting RNA synthesis and topoisomerase II (TOPO-II) [43, 44].

We previously synthesized the [Pt(*η*^1^-C_2_H_4_-OMe)Cl(phen)] precursor leading by direct reaction with DMSO, to the formation of [Pt(*η*^1^-C_2_H_4_-OMe)(DMSO)(phen)]^+^ (**1**) [21]. This complex (**1**) demonstrated a broad spectrum of antiproliferative activity toward several cancer cell lines and antimetastatic properties often superior to those exhibited by cisplatin [21–23]. Furthermore, (**1**) was also able to reduce glutathione (GSH) levels possibly as a specific effect of the drug-induced stress [23]. This result was interesting, since GSH is the most abundant antioxidant in eukaryotic cells, showing particularly high levels in mitochondria, where it contributes to redox balance through ROS detoxification and phospholipidic membrane protection [45]. Moreover, GSH mediates many other physiological reactions, including cellular signaling, cell cycle regulation, proliferation, and apoptosis [23].

On this basis, we further optimized the general structure of (**1**), by synthesizing the series of [Pt(*η*^1^-C_2_H_4_-OR)(DMSO)(phen)]^+^ {R = Me (**1**), Et (**2**), Pr (**3**), Bu (**4**)} complexes with different alkyl chain length [25]. Indeed, we observed that the variation of the alkyl chain length could improve the antiproliferative properties of these complexes with an optimal length depending on the cancer cell type. In fact, the cytotoxicity of complexes **2**, **3**, and **4** resulted higher in hepatocarcinoma cells where complex **1** was less effective [21, 25]. On the other hand, antiproliferative properties of platinum complexes seem related to molecular lipophilicity [46, 47]. Thus, the gradual modification of the Pt complex structure may lead to the optimization of antitumor activity and drug selectivity. Among the four above-described phenanthroline-containing complexes, **2** was generally the most cytotoxic [25]. For these reasons, we decided to study in this work the antiproliferative activity of [Pt(*η*^1^-C_2_H_4_-OMe)(DMSO)(phen)]^+^ (**1**) and [Pt(*η*^1^-C_2_H_4_-OEt)(DMSO)(phen)]^+^ (**2**) on three PDAC cell lines (Mia PaCa-2, PANC-1, and YAPC) and compare their proapoptotic effects with those of cisplatin. Cytotoxicity assays allowed us to verify the correlation between the compounds' hydrophobic properties and their antiproliferative activity on pancreatic cell lines with different phenotypic and genotypic characteristics [29, 48]. The cytotoxicity of phen-containing complexes was determined by SRB assay in the three PDAC lines, using cisplatin as a control. Generally, the two cationic complexes had superimposable effects on cell viability, except for MIA PaCa-2 cells where complex **2** was more effective. Meanwhile, a rapid and higher antiproliferative effect was observed for both complexes **1** and **2**, with respect to cisplatin, as shown in Figure 2. Complexes **1** and **2**, after 24 h of incubation with YAPC cells, showed IC_50_ values about 6 times lower than cisplatin, indicating their high cytotoxic effect also on cisplatin-resistant tumor lines. Moreover, the cationic complexes **1** and **2** exhibited very similar uptake profiles and cytotoxicity levels in the examined PDAC lines, suggesting that the cytotoxicity of complexes **1** and **2** on YAPC cells may be related to a high intracellular uptake with respect to cisplatin, as shown in Figures 2 and 3. Since the induction of apoptosis on YAPC cell line after exposure to cisplatin, [Pt(*η*^1^-C_2_H_4_-OMe)(DMSO)(phen)]^+^ (**1**) and [Pt(*η*^1^-C_2_H_4_-OEt)(DMSO)(phen)]^+^ (**2**), gave similar results for the two phenanthroline-containing complexes (*p* > 0.05), we only focused on complex **2** (showing slightly higher activity) for some of our further investigations.

Flow cytometry analyses demonstrated that complex **2** treatment significantly increased levels of apoptosis in YAPC cells, even more than cisplatin, as shown in Figure 4. Furthermore, [Pt(*η*^1^-C_2_H_4_-OEt)(DMSO)(phen)]^+^ (**2**) exposure caused a high accumulation of sub-G1 phase cells compared to cisplatin, which is indicative of cell death, as shown in Figure 5. The accumulation in the sub-G1 phase after treatment with cisplatin could be related to the occurrence of DNA repair system defects, resulting in altered cell cycle regulation and increased cell death [49]. We recently hypothesized a cytosolic target for [Pt(*η*^1^-C_2_H_4_-OMe)(DMSO)(phen)]^+^ (**1**), which caused a faster and greater inhibition of cell proliferation and metabolic alteration [22, 23]. In this study, differently from cisplatin, complex [Pt(*η*^1^-C_2_H_4_-OEt)(DMSO)(phen)]^+^ (**2**) demonstrated an early induction of apoptosis and cell cycle block at sub-G1 phase, thus confirming that these type of Pt(II) compounds have a cellular target different from that of cisplatin. The lipophilic cation JC-1 was also used to assess whether complex **2** induced alterations of mitochondrial membrane potential (ΔΨ_M_) in PDAC cells. The aggregation of JC-1 in the mitochondria is driven by the transmembrane potential. Yellow-orange fluorescence of JC-1 dimers is present in cell areas with high mitochondrial membrane potential, while green fluorescence of JC-1 monomers is prevalent in cell areas with low mitochondrial membrane potential. As shown in Figure 6 in control cells with intact nuclei, JC-1 aggregates inside healthy mitochondria and fluoresces red while, in apoptotic cells with fragmented nuclei, the monomeric form of JC-1 fluoresces green. In the YAPC line, progressive loss of ΔΨ_*M*_ as measured by the green/red emission pattern of the mitochondria-selective dye JC-1 was induced by cisplatin. On the other hand, in [Pt(*η*^1^-C_2_H_4_-OEt)(DMSO)(phen)]^+^ (**2**) treated cells, ΔΨ_*M*_ significantly decreased during the first minutes of incubation with Pt compound as observed by fluorescence data, as shown in Figure 6(c). The electron transport chain generates an electrochemical gradient across the inner mitochondrial membrane, which is essential for ATP production by mitochondrial ATP synthase and also allows cargo delivery into mitochondria using cations [50, 51]. It was demonstrated that various molecules with anticancer properties, including metal-based compounds, are able to act directly on mitochondria, inducing loss of membrane potential and release of apoptotic proteins [52–55]. Cisplatin may induce changes in ΔΨ_*M*_ and activate damage pathways by activating several proteins and binding to mitochondrial DNA [51, 53, 56–58]. In the present case, given the rapid reduction of ΔΨ_*M*_, the cationic phenanthroline-containing complexes could cause alterations in mitochondrial morphology and function by interacting with mitochondrial membrane proteins. Moreover, it has been reported that the membrane potential of the mitochondria in cancer cells is higher than in normal cells, resulting in the accumulation of cationic lipophilic compounds down an electrochemical gradient [55]. Therefore, the effect of alkyl chain length and related hydrophobic properties could allow these Pt(II) compounds to be readily taken up by mitochondria, a possible example of passive drug delivery.

Finally, the effects of cisplatin, [Pt(*η*^1^-C_2_H_4_-OMe)(DMSO)(phen)]^+^ (**1**), and [Pt(*η*^1^-C_2_H_4_-OEt)(DMSO)(phen)]^+^ (**2**) on YAPC cells' migratory properties were examined by 3D tumor spheroid-based assay. Culturing cells in a three-dimensional context produces distinct cellular morphology and signaling when compared to a two-dimensional culture system [59]. For example, cancer cells could show *in vitro* multicellular drug resistance when they were grown in 3D configurations rather than monolayer cultures [60]. Complexes **1** and **2** significantly reduced the migration area around the tumor spheroids, compared to cisplatin as shown in Figure 7, as already demonstrated for **1** on neuroblastoma cells [22].

In conclusion, both cationic complexes [Pt(*η*^1^-C_2_H_4_-OR)(DMSO)(phen)]^+^ {R = Me (**1**) and Et (**2**)} were found to contrast PDAC progression by inducing apoptosis and inhibiting metastatic processes. In the case of YAPC cells' response to complexes **1** and **2**, significant differences were observed compared to cisplatin, with both complexes exhibiting significantly higher intracellular accumulation within the first 24 hours of treatment, with complex **2** displaying a slightly higher intracellular accumulation than **1**. Despite the differences in cell accumulation, similar anticancer effects were observed, not related to the alkyl chain length as previously observed in other tumor cell lines [25]. These phenanthroline organometallic compounds entered YAPC cells and mitochondria very rapidly, causing a fast induction of cell death, likely due to the activation of mitochondrial apoptotic pathways [22]. It is well-established that cationic platinum complexes can be actively transported by organic cation transporters (OCTs) of the SLC22 family, enhancing intracellular accumulation and antitumor activity, with phen-related ligands generally favoring this process, as observed for phenanthriplatin [61, 62]. Therefore, the results of this study, which demonstrate a rapid intracellular accumulation of both complexes **1** and **2**, appear to further suggest an involvement of cell membrane OCTs in the cell membrane crossing of these organometallic species, thus improving their antiproliferative and antimetastatic effects.

## Data Availability

All data supporting the results are included in the article.
